# Supplementary material for: Comparative multiomics analysis of cell physiological state after culture in a basket bioreactor
Source: Sci Rep. 2022 Nov 23;12:20161. doi: 10.1038/s41598-022-24687-4 (PMC9686226; doi:10.1038/s41598-022-24687-4)
Supplement: Supplementary file 1 — Supplementary Information 1. [file 41598_2022_24687_MOESM1_ESM.zip › raw data/Transcriptomic raw data/6.Enrichment/2.KEGG/TE1vsTC1/UP/src/csab04931.html]

csab04931


- csab:103226390

- Up regulated genes

103226390(2.68260263702081)

- csab:103214788

- Up regulated genes

103214788(1.09310575924899)

- csab:103226390

- Up regulated genes

103226390(2.68260263702081)

- csab:103247816

- Up regulated genes

103247816(2.00172023185039)

- csab:103220112

- Up regulated genes

103220112(1.27838901901387)

- csab:103247816

- Up regulated genes

103247816(2.00172023185039)

Close
